# A Review of Indigenous Food Crops in Africa and the Implications for more Sustainable and Healthy Food Systems

**DOI:** 10.3390/su12083493

**Published:** 2020-04-24

**Authors:** Racheal Akinola, Laura Maureen Pereira, Tafadzwanashe Mabhaudhi, Francia-Marié de Bruin, Loubie Rusch

**Affiliations:** 1 Faculty of Agrisciences, Stellenbosch University, Mike de Vries, Merriman Ave, Stellenbosch Central, Stellenbosch 7600, South Africa; 2 Centre for Food Policy, City University of London, Northampton Square, London EC1V 0HB, UK; 3 School of Life Sciences, University of KwaZulu-Natal, P. Bag X01, Scottsville 3209, Pietermaritzburg, South Africa; 4 Centre for Complex Systems in Transition, Stellenbosch University, Stellenbosch 7600, South Africa; 5 Centre for Transformative Agricultural and Food Systems, School of Agricultural, Earth and Environmental Sciences, University of KwaZulu-Natal, P. Bag X01, Scottsville 3209, Pietermaritzburg, South Africa; 6 Faculty of Economic and Management Sciences, Stellenbosch University, Stellenbosch Central 7599, South Africa; 7 Making KOS, 7 Purley Street, Kenilworth 7708, South Africa

**Keywords:** ITFC, food system, nutrition, environment, social-cultural, economic

## Abstract

Indigenous and traditional foods crops (ITFCs) have multiple uses within society, and most notably have an important role to play in the attempt to diversify the food in order to enhance food and nutrition security. However, research suggests that the benefits and value of indigenous foods within the South African and the African context have not been fully understood and synthesized. Their potential value to the African food system could be enhanced if their benefits were explored more comprehensively. This synthesis presents a literature review relating to underutilized indigenous crop species and foods in Africa. It organizes the findings into four main contributions, nutritional, environmental, economic, and social-cultural, in line with key themes of a sustainable food system framework. It also goes on to unpack the benefits and challenges associated with ITFCs under these themes. A major obstacle is that people are not valuing indigenous foods and the potential benefit that can be derived from using them is thus neglected. Furthermore, knowledge is being lost from one generation to the next, with potentially dire implications for long-term sustainable food security. The results show the need to recognize and enable indigenous foods as a key resource in ensuring healthy food systems in the African continent.

## 1 Introduction

An erosion of indigenous and traditional foods crops (ITFCs) and agricultural production in the Global South has dramatically changed the global food system in the last 50 years [1]. Since the Green Revolution in the 1960s, agriculture has mainly focused on developing conventional cereal and horticultural crops, and as a result, these foods became more popular and replaced many locally produced crops, leaving the development and cultivation of ITFCs to be severely undervalued [2]. This transition has swept across the world so much that ITFCs, once widely used, have been replaced with lower nutrient foods, with an increase in health-related problems [1].

Over millennia, ITFCs were a main source of food for communities, but a post-colonial displacement of these foods and their food ways occurred, as they were portrayed as poor men’s food by colonizers [3]. In South Africa, research and extension has been labeling traditional leafy vegetables as weeds since the 1960s; this unfortunate label has stigmatized these crops, especially among young people [4]. It has led to a decline in the use of these wild-food resources in many communities, as well as the conveyance of knowledge associated with the plants [5].

More than 50% of the world’s daily requirements of calories and protein now come from three main staples: wheat, maize, and rice [6]. What is more, nearly 80%–90% of our total dietary intake comes from 12–20 species [7]. Although this ensures adequate calories, it inadvertently neglects the need for dietary diversity and the nutritional needs [8]. By contrast, ITFCs provide greater diversity than exotic foods, with 7000 species having been used throughout human history as food sources and multiple other uses [9,10]. Given the need for dietary diversity and ongoing concerns linked to environmental impacts like climate change, ITFCs are strategically placed to provide strategic food options that have potential to improve nutrition, increase dietary diversity and that are adapted to climate change [11].

Following the literature on mainstreaming agrobiodiversity for sustainable food systems [12], the benefits of ITFCs in enabling more sustainable and equitable food systems can be classified into four main groups:

1. nutritional benefits, e.g., nutrient density can be higher than in other foods [13];
2. environmental benefits, where for example, ITFCs can be drought tolerant in the face of climate change [14];
3. social-cultural benefits, e.g., the interaction between local knowledge and nutritional value of indigenous foods [15];
4. economic benefits, e.g., livelihoods and income generated due to the sales of ITFCs [10].

However, despite these perceived benefits of indigenous foods, their use within Africa has not been widely studied [16]. Relatively few are economically utilized [11], most notably due to factors including cultural values, human perceptions, and lack of consumer awareness about their benefits [17].

Traditional foods serve as a symbol of heritage, trademark, and culture, besides offering an important opportunity to diversify the food base. These foods are fundamental to many cultural identities of diverse ethnic groups [18,19]. It is therefore important to preserve diverse food practices, especially elements of food preparation and consumption, as this knowledge can easily be lost over a few generations. There is significant risk that the knowledge around ITFCs is already being eroded, and along with it, potentially crucial ways of living more sustainably [20]. Many examples have been reported in the African context where communities have lost their food sovereignty, losing the right to determine their own food system and its management, which has led to food insecurity [21]. This is evident in other developing country contexts; for example, Mexico, where agrarian communities have encountered numerous influences that have caused a transition from self-reliance to a dependence on the industrial food chains [22]. Similarly, in most African countries, the ethno-botany of wild food resources is poorly documented and patchy, consisting of lists of plant names, providing little to no information on their use and management [11,23].

Scholars argue that although indigenous food plants have, in the past, played an important role in the diet of African communities, the industrialization of food and formalization of markets in countries like South Africa have resulted in a decrease in the use of established domesticated wild plants and foods that had been stable for decades [24]. Balinga [25] elaborates further, stating that traditional foods have been marginalized, owing to the lack of information on the extent of their use and importance in rural economies; their economic value; reliable methods for measuring their contribution to farm households and the rural economy; lack of world markets (except for a handful of products); irregularities in supply; quality standards; storage and processing technology.

All these highlight significant gaps in the recognition of African indigenous foods. Although the World Trade Organisation (WTO) states that “Indigenous agriculture and biological resources are vitally important to the economies, cultures, environment, food security and livelihoods of sub-Saharan Africa” [26], there is a scarcity of data on ITFCs, particularly in sub-Saharan Africa, and the demand for ITFCs cannot be properly realized or investigated without these crucial data [16]. Work done by researchers such as Mbhenyane [16] suggests that there may be great potential for African food systems and its food security if indigenous plants were studied more extensively and included more often as mainstream foods. It is clear that further research in this area is imperative, more studies are required to contribute new knowledge to science, hence increasing awareness on traditional uses and management of ITFCs [11]. As such, in this paper we aim to provide a synthesis of existing information on ITFCs in Africa in order to bolster the argument that these foods can contribute to food and nutrition security on the continent and to identify gaps where more research is urgently needed.

## 2 Methods

The research design consisted of a systematic literature review of current academic and grey literature, providing an overview of the knowledge and gaps within the field of indigenous foods. The methodology adapted for this review was based upon the work of Pereira [27].

The following databases were used for the peer-reviewed articles: SUN Search (Stellenbosch University Online library, which comprises a collection of ten key domestic and international databases), South African Theses and Dissertations (ETD) portal, Scopus, and Google Scholar. A comprehensive inquiry of keywords was set around the topic for articles published from 2008 to 2019. The topic criteria included indigenous (and/synonyms) food crop, South Africa, Africa, in the title, abstract, keywords, introduction, or conclusion.

The inclusion criteria included literature that showed connections to:

1. nutritional value/benefits/challenges;
2. environmental value/benefits/challenges;
3. economic value/benefits/challenges;
4. social-cultural value/benefits/challenges;
5. food system.

The exclusion criteria included:

1. articles outside the time period of the study (2008–2019);
2. articles on other characteristics of ITFCs (morphology, physiology, disease pathology, etc.) not related to the inclusionary themes; and
3. publications where Africa and/or African countries are not the main point of focus.

Subsequently, the abstracts were reviewed, and final articles were selected based on the abstract, providing they met the said criteria (See Table 1). Further articles were selected if they provided information on the role of ITFCs in the food system, and/or food and nutrition security, and/or in communities. Publications that did not meet these above set criteria were excluded from the selection. Articles on livestock and marine animals were also excluded, as this review focused on crops, however future reviews should definitely also take animals into account.

A few organization websites were chosen for the grey literature searches. This included Biodiversity International, the Food and Agriculture Organization of the United Nations (FAO), the World Trade Organization (WTO), the International Fund for Agricultural Development (IFAD), and the World Bank. The addition of grey literature provided extra data that was invaluable both as literature and as a way to obtain relatively unknown literature.

The inclusion and exclusion criteria were applied to the initial results (*n* = 11,444), which were then reduced to (*n* = 267). The inclusionary literature was then cross-referenced to further extrapolate literature within the given parameters and new literature was extrapolated (*n* = 38) from the reference list of literature found. Therefore, with the use of cross-referencing and requesting of further data and literature from authors and papers recommended due to proximity, the end result, a total of 305 literature documents were chosen for inclusion in the final database. Table 1 below illustrates the databases and websites searched, search terms, limits that were set in terms, and number of papers subsequently included.

To document the benefits and challenges with regards to ITFCs, the literature gathered was categorized into four key themes of focus, namely nutrition, environmental, economic, and social-cultural. About 41% of the included studies focused on the nutrition aspect of ITFCs, the majority of which were studies on the nutrient composition of the crops. A total of 23% highlighted the environmental impacts of ITFCs, 21% of the articles were focused on the socio-cultural aspects of ITFCs, while 15% were on the economic characteristics of ITFCs. These categories were chosen since they encompass the key elements related to the framework for sustainable food systems. According to the FAO, “a sustainable food system is a food system that delivers nutrition for all in such a way that the economic, social and environmental bases to generate food security and nutrition for future generations are not compromised” [28]. The theme “environmental” looks at the impact of ITFCs on the natural environment conserving biodiversity and agricultural ecosystems. The category of nutrition looks at the promotion of the foods themselves and what nutrition they can provide, as well as the contribution towards people’s health. The social-cultural theme looks at the benefits of ITFCs to society, people’s behaviour towards ITFCs, and social concepts around ITFCs, while “economic” looks at the role ITFCs in improvement livelihoods through the improvement of economic viability [29]

## 3 Situating the Review in the Existing Literature

A definition of indigenous foods is elusive due to the encompassing variety of foods, fruits, leaves of trees, leafy and root vegetables, and herbaceous plants that it includes [30]. *Traditional* food is differentiated from *indigenous* foods by traditionally having been eaten within the last few centuries, whereas indigenous foods are not defined by a set time period [31]. According to Ayanwale [32], indigenous food is defined as foods originating in a specific bio-region in conjunction with foods that were introduced into the country and are now recognised as indigenous due to their being integrated into the local food culture. Mbhenyane [16] refers to indigenous foods as non-commodities (something for which value is not recognized) that form part of a large portfolio of genetic, agroeconomic, economic, social, and cultural factors. Others have noted that ITFCs usually exist independently of direct human action, illustrating their adaptation to the environment and resilience without human interference [33].

According to Bharucha and Pretty [10], ITFCs can be envisioned as foods existing in a continuum from entirely wild foods to semi-domesticated foods, where human intervention is seen ranging from harvesting, propagating, to transplanting. These two parts of the continuum are described in two categories of classification: conventional (semi-domesticated) and less conventional (wild) indigenous foods [30]. Conventional indigenous foods are foods such as sorghum, cowpeas, and sweet potatoes, whereas less conventional indigenous foods are those such as amaranth, pumpkin leaves, and calabash [30].

ITFCs are often referred to interchangeably with wild foods and “orphan crops” or “neglected, and underutilized species” (NUS) and are viewed as secondary crops [34]. NUS are defined in two ways by the International Plant Genetic Resources Institute (IPGRI) where *neglected* is referring to the status of these foods as “minor crops”, yet still being utilized to a small degree for rural subsistence farming. *Underutilized* refers to a domesticated plant species that has been used for millennia and grown intensively for its properties as foods, fodder, and fiber. However, these foods have been reduced in importance particularly in usage and due to supply constraints. Often indigenous foods will be relegated to an NUS status [7,34,35]. A new terminology has been coined for ITFCs and NUS, namely, *new or future crops*. New and/or future crops refer to crops that previously did not have noteworthy industrial importance due to low levels of utilization, but nonetheless illustrate new potential to instigate novel value chains if there is a significant increase in development and research actions [36].

It is fair to ask why Africa's indigenous crops are not better known. According to the National Research Council [37], the reasons can be attributed, at least in part, to several unjustified perceptions and misperceptions that are clouding the world’s vision of Africa’s native foods, such as inferiority of ITFCs, which has caused a strong inclination to consider the introduced or exotic crop superior and the native crop obsolete and unworthy of further development. In West Africa, for example, Asian long grain rice has replaced African rice as a staple food. This is illogical, ill-conceived, and holds crucial economic consequences for any country. In Nigeria, it is reported that Asian rice valued at N2.34 billion was imported into the country between October 2015 and March 2016 [38]; one can only imagine how the economy will improve if these monies were to circulate within the country. Another misconception, which is perhaps the most frequent comment often made about ITFCs, is that they produce inferior yields [37] compared to conventional crops. Ayanwale et al. [32] further explains this point that ITFCs are underutilized largely due to the low production by smallholder farmers.

On the other hand, ITFCs are being partly maintained because of their social-cultural significance, their easy usage, and their importance for the subsistence of local communities [32] particularly in terms of food and nutrition security and income generation [39]. ITFCs would therefore occupy a niche within the local ecology and markets in the community if better utilized. This highlights the importance of the need for establishing a viable supply and demand chain as well as a value chain for ITFCs [29]. However, due to a lack of modern and industrialized markets for ITFCs, there is a tendency for merely little development or investment both socially and scientifically into the benefits and utilization of ITFCs within Africa [16,40]. There is a further knowledge gap between the possible ITFC varieties that can be used for consumption and the actual varieties being consumed due to decreased use and limited knowledge of these foods [41]. Even though studies show a lack of adequate usage of indigenous nutrient sources, FAO estimates that about one billion people globally use wild and/or indigenous foods in their diets [10,42].

The active incorporation of ITFCs into diets can be seen as forming part of an alternative food system network. Alternative food networks (AFN), by their nature, challenge the status quo of ethical and ecological bases of transnational agri-food supply chains [43]. AFN is defined as the “rejection of the global, industrial, environmentally degrading conventional food system” [44]. AFNs “offer a vision that people, by eating differently, can change the worlds of food…”. AFNs exist in many diverse forms, from fair trade cooperatives to local farmers markets and movements such as La Via Campesina [45]. The rise in alternative food systems, community gardens, and localized food use is described by Wiskerke [46] and Colin [43] as being due to a generalised disenchantment with the corporate-led global food system: “Growing food… opens a space to challenge the mainstream food system by offering a more equitable, ecologically sustainable and potentially socially empowering alternative” [43].

## 4 Findings

More than 90% of all ITFCs in Africa originated in, or were ancient introductions to the continent, with only 8% recently introduced. Moreover, there are 400 well-defined plant species that encompass 53 botanical families, primarily used as vegetables in Africa [47]. This illustrates the richness of ITFCs in Africa as well as their potential. More ITFCs have been utilized and cultivated in larger quantities in the rest of Africa than in South Africa. Although Bvenura and Afolayan [2], citing from various literature sources, reported a total of 103 species from 33 families, most of these crops are eaten sparingly and are simply collected from the wild, and no effort is made to specifically cultivate them for food. The poor level of utilization of ITFCs in South Africa has been attributed mainly to the historical context of the country, where the Apartheid regime restricted access and relocated people from their ecological environment, thereby limiting the continuation of the use of ITFCs and their associated knowledge. The ITFCs which are actively cultivated for markets in South Africa are Rooibos, Buchu, Pelargonium, and herbs [48]. Currently, the South African food composition database, which comprises a total of about 1472 food items, has only 21 indigenous leafy vegetable food items from 12 species of indigenous vegetable species [2,49]. This indicates a gap in the literature for South Africa, not only on the nutritional benefits but on the other potential benefits of these foods as well. However, in order to include more traditional food in the database, a majority of the populace needs to start by making them a part of their diet.

### 4.1 Nutritional Benefits and Challenges

ITFCs have a host of desirable traits. Many of them are richer in protein and other nutrients than popular non-native crops [50]. They have been compared to “super foods”, in that they offer healthy, accessible, and affordable nutrient dense alternatives [51], which can contribute to addressing gaps in nutrition. ITFCs are good sources of macro and micro-nutrients for human consumption; many traditional or indigenous vegetables are characterized by a high nutritional value compared with global vegetables like tomato and cabbage [52]. Most notably, many are sources of vitamins and macro and micro elements with the potential to provide them to children and adult at levels exceeding those recommended by the World Health Organisation (WHO) [53]. According to the WHO, “a healthy diet helps to protect against malnutrition in all its forms, as well as against noncommunicable diseases (NCDs) such as diabetes, heart disease, stroke and cancer”. To achieve this, at least 400 g (i.e., five portions) of fruit and vegetables per day (excluding potatoes, sweet potatoes, cassava, and other starchy roots), as well as legumes, nuts and seeds, and whole grains is recommended for adults [54].

There is no doubt that ITFCs can provide the full nutrient requirement, with over 400 documented species of vegetables alone and countless species of fruits, grains, pulses, and tubers. ITFCs also have the added benefit of creating diversification of foods grown and increasing the likelihood of diversified diets. Local biodiversity of food is an important contributor to wholesome, nutritional diets. Even small quantities of nutritionally dense foods may be of significant benefit when the rest of the diet is void of nutrients and minerals. This is especially the case in food diets based largely on starchy staples [13,55]. Even more importantly, some of these crops have the potential for year-round accessibility, with significant overlap of crop abundance at times of acute food and nutritional scarcity [56], while some crops have multiple edible parts in one plant. A notable example is the Aizen plant found in the Sahel region—t he seeds, fruits, roots, and leaves are all edible parts of the plant [37]. Another is the African yam bean, one of only two plant species in the world known to produce both a legume and an edible tuber [57]. Implementing ITFCs can therefore have a positive effect on the reduction of the cost of daily required diet, as well as making up for nutrient shortfall [58]. By including ITFCs, local biodiversity can contribute to a wholesome and nutritionally rich diet. Appendix 1 and 2 provide lists of notable traditional foods compiled from literature, along with their nutritional benefits and the geographic distribution of the crops.

ITFCs have broader nutritional application and benefits, including the potential for bioactive compounds that can contribute to antioxidant activity in the body. Mbhenyane et al. [59] expounds that ITFCs contain phytochemicals and antioxidants that are linked to protecting against the development of diseases through nutraceutical effects. ITFCs have been a source for traditional medicine for many years. Even today, the seeds, leaves, bark, and roots of various plants are used in traditional, complementary, and alternative medicine (TCAM) in many African cultures. In fact, the WHO estimates that a considerable number of people in Sub-Saharan Africa rely on TCAMs to meet their primary healthcare needs [60]. Nowadays, thanks to pharmacological studies and ethno-botanical analysis on various traditional crops clearly demonstrating their pharmacological activities and contribution to people’s health, ITFCs are playing a crucial role in modern medicine, gaining the attention of many pharmaceutical laboratories [61]. For example, crops like rooibos and moringa have gained international acclaim for their therapeutic potentials in targeting many diseases, like diabetes, cardiovascular diseases, including hepato and nephro-protective activities [62–64]. Some species of traditional green leafy vegetables like *Telfairia occidentalis*, *Vernonia amygdalina*, seeds like *Garcinia kola*, and pulses like Bambara groundnut (*Vigna subterranea*) are being researched for their potential pharmacological and biological activities on diseases like diarrhea, anaemia, ulcers, and cataracts, diabetes, and even fertility, based on their suspected antioxidant and anti-inflammatory, anti-proliferative properties [65–67]. Table 2 provides a list of the broader benefits of ITFCs.

In terms of constraints to use, there are various factors that challenge the use of ITFCs as food. Some of these constraints are based on empirical studies, while some are based on analysis on the food’s nutrient composition.

1. ITFCs can have a bitter taste and as such discourage people from consuming them [68]. Westernization of the African diet has most likely affected people’s palate and food habits such that traditional foods are no longer enjoyable [2];
2. Some ITFCs are difficult to process (e.g., to dehusk and thresh). Some crops can take hours to prepare, and some do not change through preparation, e.g., they maintain a peculiar color, even after cooking;
3. The chemical, nutritional, and toxicological properties of ITFCs, for example, the presence of anti-nutritional factors (ANF), can have a severe nutritional impact that may limit food application. These compounds are known to interfere with metabolic processes such that the digestibility and bioavailability of nutrients are negatively impacted, with potential risk of toxicity [69,70];
4. There is a lack of recipes to follow as guidelines [71], maybe resulting from a lack of enthusiasm to learn about them, or there is no one to teach the younger generations about methods of preparation and methods of preservation [2,5,72].

### 4.2 Environmental Benefits and Challenges

It has been reported that in 2011, a mere three crops—wheat, rice, and maize—covered 555 million ha, or 40% of all arable land globally [81]. This reliance on a handful of major crops has inherent agronomic, ecological, nutritional, and economic risks and is unsustainable in the long run, especially in view of global climate change [52]. It is widely accepted that climate change has a major impact on agricultural production systems and threatens yield and crop sustainability [82]. The sub-Saharan region has been categorized by the Intergovernmental Panel on Climate Change (IPCC) as one of the regions that is most vulnerable to climate change and variability. This is further aggravated by the fact that globally it is also one of the regions with the least adaptive capacity to climate change [7,83,84]. This is of immense concern and may provide the right push to tap into alternative food networks (AFN), to create new food systems and cropping structures that will promote greater diversity. This will build spatial and temporal heterogeneity in cultivated crops and enhance resilience in agricultural cropping systems [85]. Exotic crops often lack resilience in the foreign systems in which they are grown, indicating that indigenous crops such as barley, millet, and sorghum, which are generally more climate tolerant [86] and adapted to diverse ecological niches [87], can be more resilient to changes in climate and environment than exotic crops [88].

ITFCs enable new narratives on farming methods and techniques for improving environmental conditions. Consequently, they provide valuable reservoirs in terms of crop genetic assortment as well as biological diversity with regards to farming operations [80,86]. Some of the ITFCs can be applied to various farm management systems to increase the natural fertility of the soil. This may be through nitrogen fixation (e.g., legumes) or increasing organic matter through higher populations of soil microorganisms. They provide other environmental benefits, such as improved soil and water conservation, better nutrient retention, lowering of soil temperatures, and a source of ground cover, which can serve as a means to reducing soil erosion as well as earning carbon credits; some have the potential to purify water, assisting with ecosystem restoration overall [7,55,89]. Other ITFCs contribute to food and nutrition security by being adapted to extreme climatic and edaphic conditions, such as high soil salinity [90], and are able to grow even in drought-stricken areas, e.g.m Amaranth, Fonio, Millet [7,91], and Slenderleaf (*Crotalaria brevidens*), which has been shown to be particularly hardy during droughts due to its quickly established taproot [50].

ITFCs are usually easily cultivated and require fewer inputs than their exotic counterparts, and some can be grown under marginal conditions due to their tolerance to poor soils, fires, insects, and pests. Researchers attribute this tolerance to the fact that ITFCs have not passed through the genetic bottleneck of domestic cultivation, and thus have greater genetic assortment to deal with adverse effects [30,92–95]. This benefit may extend even to exotic crops if integrated in cropping systems with traditional crops. Experiments revealed that the diversification of the soybean cropping pattern with traditional grains can result in a significant decrease in the use of agrochemicals and fossil hydrocarbons without having a negative impact on yield and profitability [96], hence reducing the cost of producing crops, while benefiting the environment. Some ITFCs, like a certain strain of indigenous barley, have been shown to have up to 25% and 61% higher yields than conventional varieties [97], contrasting the generally held view that many ITFCs have low yield. Furthermore, the integration of native perennial plant species, such as trees, different grasses, and mixtures of multiple grassland species, into the agroecosystem can generate large amounts of biofuel feedstocks and at the same time increase soil carbon storage and decrease nitrogen leaching into the ground [52,98]. Table 3 provides additional environmental benefits of ITFCs.

Several reasons can be put forward to explain the neglect of ITFCs by the wider climate change community. First, the number of species involved is vast, and on the other hand, increased mechanization demands and expectations of the modern food supply chain is causing farmers to focus on fewer and fewer crops [99]. Second, many species are poorly known and/or are only used locally [100]. Matenge et al. [101] notes more reasons for their neglect, due to issues of cultivation and too many related environmental challenges. As Cernansky [50] puts it, “a main focus has been basic problems such as difficulties with germination and a lack of information about how best to store seeds”. Indigenous vegetables are not up to modern farming standards for characteristics such as uniformity of seeds and yield. Seeds for most of the ITFCs are not readily available on the market, which indicates that this might be a potential challenge to scaling in the future [102,103], especially in terms of a loss of genetic diversity, so there is a lot of catching up to do. Another challenge is that modern farming systems that use large amounts of chemicals (herbicides, pesticides) and pollutants from industries may contaminate the soil, which can have a negative impact on naturally growing ITFCs nearby [104]. Although the foraging of wild foods or ITFCs illustrates the need for an alternative food source, it may become unsustainable in the long term, largely due to population pressure [9,104]. Moreover, over-harvesting decreases availability, influencing the predictability of ITFCs availability, and therefore food security of those who use it consistently [10,105].

### 4.3 Socio-Cultural Benefits and Challenges

ITFCs have high social value, hence, the main socio-cultural benefit that emerged from this review was “reconnecting with one’s roots”. In many cultures of Africa, ITFCs are often the highlight of certain rituals, ceremonies, and traditional events. For example, the “new yam” festival in East Nigeria is culturally rooted in ancient agrarian Igbo culture, is held to herald the harvest season, and provides an opportunity for a social gathering of the community [110]. Likewise, in Namibia, ITFCs and their products are presented to guests at weddings and other ceremonies as part of celebratory rituals [111].

ITFCs act as versatile foods, contributing many functions, from food for both humans and animals, as well as being a connector to the land and creating a space for adaptive management to take place. Furthermore, ITFCs could aid in creating new policies to redress past imbalances within society, such as those that resulted during Apartheid, with many feeling displaced from their places of residence. Thus, the rehabilitation of ITFCs may create a sentiment of imbalances being addressed [112], reaffirming the identity of both the individual and the community. The emphasis here is the culture inherent in the production and use of ITFCs, which, according to Bacchetta et al. [80], establishes a living link with the land, “a keystone of culture”. As Kuhnlein et al. [104] puts it, reaffirming the connection between nature and culture, mind and heart, is the foundation for cultural identity and a basis for social support networks. Table 4 highlights additional social benefits of ITFCs.

The most frequent challenges of ITFCs identified were “a lack of knowledge in identifying which indigenous foods are edible”, “a lack of knowledge in general about these foods (finding seeds, how to cultivate and use them)”, “inadequate nutrition-based testing of indigenous foods”, “lack of market access”, and negative perceptions of their status. Moreover, a major challenge to the use of ITFCs is perception of them and the difference between intention to use and actual practice. ITFCs are associated with poverty (poor man’s food) and low self-esteem, while many experience them as old-fashioned food, or as weeds. Younger generations in particular show reluctance to learn about ITFCs and associate negative attitudes towards them [3,14,30,78]. The social perceptions of the usage of ITFCs therefore seem to hamper the progress and marketability of these foods [8,14,82,113]. This has also been seen elsewhere in the world and the challenge has been framed as: “promoting the consumption of wild foods to counter negative perceptions and attitudes to local, traditional foods...” [114]. As Mabhaudhi et al. [115] stated, “each time people choose food, they bring their past food choices, events, experiences, thoughts and feelings as well as historical context to the fore”. This was investigated by Gakobo and Jere [103], who did a study to identify consumer attitudes as the strongest predictor of consumption intention in Nairobi, Kenya. The findings illustrate that the intention to consume ITFCs is high, and so Gakobo and Jere [103] determined that the consumption of ITFCs should increase. However, when this was correlated with people using ITFCs, it was found that the use of ITFCs was actually decreasing. They concluded that for this to change, marketing practitioners and policy-makers will need to understand the intention and behaviour of potential consumers of ITFCs [103]. Most communities and stakeholders thought that the frequency of use has declined in part because “…modernisation has led people to perceive indigenous foods as inferior” [20]. Zobolo et al. [116] suggested that the perception and acceptability of ITFCs will change with large scale cultivation of ITFCs.

Further concerns are the preservation of ITFCs. During times of low availability, there has been some experimentation on how to preserve ITFCs. van der Hoeven et al. [117] found that storage of ITFCs (*Amaranthus* spp., *Cleome gynandra*, *Cucurbita maxima, Vigna unguiculata* and *Beta vulgaris*) was accomplished by washing, being made into a paste, shaped into a ball, and dried in the sun, followed by being stored in sacks, as a supplement to just blanching the foods. The dried ingredients can be boiled to accompany a starch dish instead of being boiled as harvested. However, many have used different techniques, such as supplementing ITFCs with retail foods or using ITFCs and peanut butter together to create a soup [78]. Some ITFCs are processed into jams, pickled preserves, liquors, beer, wines [118,119]. Some indicators show that there may also be a lack of incentive to grow ITFCs in the urban setting [101].

### 4.4 Economic Benefits and Challenges

Many traditional vegetables species, such as amaranth, edible rape, cassava leaves, garden egg, and jute mallow, are of considerable commercial value; their sales can make a significant contribution to household income and livelihood [10,52]. In many countries, the processing, distribution, and sale of ITFCs is carried out largely by small holder farmers in informal food markets [127]. Their consumption is influenced by price, culture, seasonality/availability, accessibility, and may be a factor hindering their cultivation on a larger scale. It may limit their accessibility in both the formal and informal markets [101]. Regardless of this, ITFCs are becoming more visible in the formal sector. According to Weinberger [128], selected traditional vegetables are becoming an increasingly attractive food group for the wealthier segments of the populace in East Africa and South East Asia. These foods are slowly moving out of the underutilized informal market category into the commercial mainstream. In fact, Weinberger [128] argues that traditional foods are not at all underutilized as commonly thought, but rather are undervalued. Authors such as Mushita and Thompson [129] noted that African farmers have the farming techniques and systems already in place to sustain ITFCs and biodiversity. They argue that in the midst of increasing climate change events and policy persuasion, the international calls to mitigate and adapt with genetically modified organisms and industrial farming methods is unthinkable. Rather, alternative methods of cultivating and using food are the solutions to the current food system paradigm, and according to Kikuchi et al. [130], consideration should be given to indigenous food markets—see Table 5.

ITFCs are generally perceived negatively on the global market, though may be positively influenced with proper packaging, improving their acceptability and preference for consumption [51]. Ramadan [131] stressed that many traditional fruits can be processed into value-added products, like beverages, juices, jams, and nectars for international markets, where the exotic character of such products as well as their nutritive value could be appreciated. For example, Amarula, a cream liqueur made from the fruit of the Marula plant, is a proudly South African export product which is rated one of the best-selling cream liqueurs in the world [132].

ITFCs represent a plethora of untapped resources which have immense potential for export. This could also translate into increased economic growth and wealth for a country. However, it will not be an easy feat to achieve this. In order to fully harness and maximise the economic potential of ITFCs, public and private sector partnerships have to prioritize the identification of high potential crops and stimulate market demand for them, both locally and for export [133]. Policy interventions are critical to ensure mechanisms are in place to support both production and access to market. Institutional and policy changes are needed to empower farmers with the capacity to improve innovation, adopt appropriate sustainable technologies, gain access to financial services, and make better use of inputs to increase productivity [134]. Perhaps there is no better example of this than the Presidential Cassava Initiative (PCI) launched in 2001 in the cassava-producing countries of West Africa, which aimed at generating 5 billion dollars of value-added cassava exports annually [135]. This initiative changed the status of cassava from a crop cultivated as food for domestic use to an export and cash crop [134]. “In the current context of unpredictable oil prices and weakness in global financial and economic systems, and one in which climate change is expected to have an ever-increasing impact on agricultural production.”, the economic challenges of ITFCs must be investigated [136]. However, these kinds of campaigns must also be undertaken with caution, as the Nigerian cassava bread initiative showed when its attempt to substitute cassava flour for imported wheat flour foundered due to a lack of market [74].

Food tourism is another channel through which ITFCs can have a positive influence on the economy. In countries like South Africa, the established wine tourism industry could serve as a conduit for establishing ITFC through food tourism as the local gastronomic experience becomes marketable. Consequently, the increase in production of ITFCs can result in employment, enhancement of household income, creation of business centers based on food processing cooperatives, and raised tax revenue [137]. ITFCs could also improve access to the health food market by providing cost competitive components. ITFCs also link people to indigenous food systems and knowledge, thus reinforcing the value of ITFCs while connecting nature and culture. Increasing autonomy may result from the use of the communities’ own seed bank and from communities joining together to create food tourism around the ITFCs grown in the area. See Table 5 for more economic benefits of ITFCs.

However, there are many economic challenges in the cultivation and use of ITFCs: the lack of available land in both urban and rural areas creates a deficit in land for exotic crops as well as for ITFCs. With the increase in globalization trends of modern farming and the influence of external market forces, the farming of ITFCs has become a costly endeavor. Since prioritization of commodity exports can divert resources and land [138], an increase in economic opportunities for a restricted number of commodity exports could lead to the marginalization of local agrobiodiversity [136]. Local market trends are often influenced by wider global tendencies, such as the continuing rise in industrial farming and volatility of the import/export markets in the value chain. This leaves the market for ITFCs significantly underdeveloped, resulting in ITFCs rarely or infrequently being stocked in supermarkets. Maundu et al. [139] refer to the change of food ways “in many countries, because of globalization, modernization and urbanization, with traditional foodways being abandoned for western style foodways”.

## 5 Recommendations and Conclusions

It is clear from the synthesis that ITFCs provide a host of benefits to sustainable foods systems in the African continent as a whole. This is illustrated by their potential to promote resilience within the food systems and enhance food and nutrition security. As a prime connector between people and their environment, ITFCs have an important role to play in the achievement of the global objectives of the Sustainable Development Goals (SDGs), thus ensuring social, economic, and environmental sustainability. This is characterized by their:

1. richness in terms of provision of healthy, nutrient dense foods, that meet nutritional requirements and promote healthy diets. This relates to SDGs 2 and 3, which are ensuring zero hunger; good health and wellbeing;
2. capacity to enhance resilience in the ecosystem by promoting genetic diversity and enhancing environmental preservation in view of climate change. This is in line with SDGs 13 and 15, which deal with combatting climate change and its impacts and protecting, restoring, and promoting terrestrial ecosystems;
3. aid in income generation to improve livelihoods for individuals and potential for profits for economic growth. This adheres to the SDG 8 themes, which are sustainable economic growth, decent and productive employment for all;
4. social value, reduced inequality, as it relates to self-worth and dignity, including a sense of belonging and connecting to one’s roots. This follows closely SDGs 10 and 12: reducing inequality in terms of gender inequality, and inequality within and among countries.

However, this contribution is being undermined due to their underutilization within the food system. Some of the factors identified that subvert the use of ITFCs include:

1. Perception of ITFCs and intention to consume: there is an overall negative attitude towards ITFCs, as they are often linked to poverty, or consumed as a last resort. This is often exacerbated by the fact that they are dismissed by the modern food system as old-fashioned foods, and disregarded by conventional agriculture as weeds [3,4];
2. Diminishing knowledge base: in most cultures in Africa, knowledge of ITFCs often resides with the older demographic and is usually passed through word of mouth. Without a system for documentation, this knowledge this is being lost from generation to generation [141]. Furthermore, the younger demographic, in particular, shows reluctance towards learning about ITFCs, possibly because of the negative attitudes associated with them;
3. Degradation of ecosystems: the spread of agriculture and the homogenization of agricultural land and crops have led to the loss of ITFC species due to the degradation of ecosystems increasingly limiting the access, availability, and use of ITFCs;
4. Rapid urbanization, industrialization is changing eating patterns and nutrition trends. Rural-to-urban migrants tend to shift from traditional food ways, towards more convenient and simplified foods [142].

Therefore, in combination with a steadily rising increase in urbanization in conjunction with climate change, the way forward to secure food and nutrition security lies in alternative food pathways. Diversifying diets in Africa with ITFCs is a sustainable way in which to supply both micronutrients and essential nutrients to fight off malnutrition and the associated health problems, particularly for poor households [47]. Mbhenyane [16] stated that “underutilized indigenous crops provide an opportunity for incorporation of alternative sources into… the food system”.

The food system as a whole is experiencing greater possibilities for alternatives than previously. Alternatives such as ITFCs and their benefits across the range of environmental, economic, social-cultural, and nutritional aspects are more than a ‘conventional’ alternative (an alternative which is based on solving a problem within the system as opposed to seeing the system itself as a problem). Instead, they might be seen as an alternative that addresses a multitude of problems by embracing social justice [143]. Embracing social justice is necessary to develop and mitigate strategies from grassroots-level up. This includes addressing poverty, food insecurity, inequality, and social exclusion within the given food system in order to broaden the benefits of ITFCs [106]. In the book ‘A culinary journey of South African Indigenous foods’ the former Minister of Arts and Culture of South Africa, Paul Mashantile, notes that “we hope to bring to the fore nutrition as well as social-cultural benefits that can be derived from the consumption of indigenous foods and drinks” [144].

To fully harness the richness of ITFCs, this review puts forward three recommendations aimed at alleviating the challenges facing ITFC’s and unlocking their vast potential.

Firstly, the current lack of awareness of ITFCs, their affordability, and multitude of benefits offers the potential for successful intervention in the food system [14]. It is vital to equip stakeholders with knowledge regarding the appropriate use and benefits of ITFCs. As Smith [145] puts it, revitalizing indigenous food systems and the knowledge accompanying them is imperative in fighting malnutrition and micro-nutrient deficiencies on the African continent. We therefore recommend that the Government, in collaboration with non-governmental bodies, community leaders, and knowledge holders of ITFCs need to come together to devise strategies and mechanisms for promoting awareness of indigenous knowledge about ITFCs among the populace. This includes the importance of the production and utilization of traditional food resources for food-security, nutrition, and income generation, as well as their role in enhancing biodiversity. It should especially target the younger generation and urban demographic. Triggering behavioral change requires strong awareness-raising, so information about ITFCs might be included in the curriculum for primary and secondary school pupils. Periodic agricultural fairs showcasing ITFCs could be set up within city centers and informative public service announcements could be disseminated via all types of media, targeting, for example, hospitals and health centers.

Secondly, in several instances ITFCs are simply collected from the wild, and no effort is made to specifically cultivate them for food. With a view to being careful to avoid over-exploitation of ITFCs, Borelli et al. [95] suggests that farmers as well as related movements should be actively engaged in the domestication of these plants. As the production of ITFCs is often limited by access to quality seeds, the Government should work alongside them as well as the scientific community through research and extension services:

a) to provide quality seeds and set in place systems for continuous seed production;
b) to develop improved varieties of ITFCs through plant breeding and biotechnology;
c) to create awareness about established indigenous farming practices as well as enhanced cultivation practices of ITFCs;
d) invest in research on affordable processing to add value to the crops once harvested.

Finally, according to Kuhnlein et al. [104], indigenous food production, harvesting, and cultivation require the support, cooperation, and collaboration of government to practice a different management system to sustain alternative sources of food. It is not only the role of governments to alleviate the unsustainability of the food system, but also the food system in itself must “shift from being part of the problem to becoming a greater part of the solution” [146]. The Government should give ITFCs the same priority and support accorded to exotic food crops, in terms of:

a) formulating nutrition and food policies that seek to improve consumer demand and preferences for ITFCs;
b) further developing agricultural policies that address the production, trade, and marketing of ITFCs;
c) having improved interactions with indigenous foods value chain actors, including the farmers, traders, and the researchers. In this way, the food system may be strengthened to enable ITFCs to become more mainstream as resilient and sustainable alternatives or supplementary foods resources;
d) encouraging the use of agro-technology in the processing and packaging of ITFCs in ways that make them more attractive while maintaining (or possibly increasing) their nutritional value.

This synthesis of the literature has demonstrated the richness of ITFCs on the African continent and highlights the potential role that these crops can play in developing a more sustainable and healthy food system for the continent. However, for their full potential to be harnessed, the research agenda needs to invest much more time and effort in understanding these crops better. Furthermore, there is also a critical need to shift perceptions of these crops away from negative, colonial connotations towards an embracing of how their diversity and the multiple knowledges associated with them can be a critical source of resilience for the global food system.

## Supplementary Material

Appendix

## Figures and Tables

**Table 1:** Electronic databases searched.

Search Engine	Phrase Particular to Index Terms	Delimiters	Results	No. Included
Peer-reviewed Literature				

SUN Search	“Indigenous food crops” “South Africa”	2008–2019	572	66
	“Indigenous food crops” “South Africa”			
Google Scholar	“neglected and underutilized”	2008–2019	1740	135
	food crops “Africa”			
	“traditional food crops” “Africa”			
Scopus	“Indigenous food crops” “Africa”	2008–2019	121	12
South African National ETD (e-theses and Dissertations) Portal	“Indigenous food crops” “South Africa”	2008–2019	6573	17

Grey Literature				

Biodiversity International	“indigenous food crops Africa” “Underutilized food crops Africa”	None	1360	37
World Bank	“Indigenous food crops Africa” “Benefits of indigenous foods in Africa”	None	500	7
The International Fund for Agricultural Development (IFAD)	“Indigenous food crops Africa”	None	11	7
World Trade Organisation (WTO)	“Indigenous food crops Africa”	None	467	3
Food and Agricultural Organisation (FAO)	“Indigenous food crops Africa” “Food system”	None	100	21
Cross Reference		2008–2019		38

**Table 2:** Broader nutritional benefits of indigenous and traditional foods crops (IFTCs).

Broader Nutritional benefits of ITFCs			

Description	Findings	Location of Study	Reference
Source of gluten-free flour	An example of this is *Eragrostis curvula*, Amaranth, Cassava	Eritrea and Ethiopia	[73–75]
Some ITFCs are important sources of water	Especially during dry months and places with a lack of surface water	Sub-Saharan Africa	[7]
ITFCs improve body functions and have nutraceutical effects	Drug metabolism, stimulation of the immune system, and boosting tissue generation, lowers the frequency of diet-related diseases, ITFCs have bioactive compounds and contribute to antioxidant activity	South Africa	[8,59,76]
ITFCs have the potential to be applied with various technologies to deliver probiotics	Probiotics, which are live microorganisms conferring health benefits to the host when consumed in adequate amounts	Southern Africa	[77]
Once-off increased cost of crop diversification of ITFCs would be significant	Beneficial in comparison to supplementation (drug treatment) and fortification	Africa	[10]
ITFCs in conjunction with new farming techniques create stronger food production systems	Increased accessibility in combination with indigenous knowledge of nutritional properties, may lead to improved food security	South Africa, Chad	[78,79]
Potential anti-bacterial products	Could theoretically be exploited in the search for novel antibiotics	Africa	[55,80]

**Table 3:** Environmental benefits of ITFCs.

Environmental Benefits of ITFCs			

Description	Findings	Location of Study	Reference
Environmental plasticity	This allows ITFCs to be planted and harvested at any time of the year	Sub-Saharan Africa	[7]
Improve urban environmental conditions, a means to supply urban dwellers with fresh food with a low carbon footprint	Create urban greening and suitable land-scaping, integration of the garden into modern civilization	South Africa	[106–108]
Can be cultivated in marginal land spaces	Adopting a more diverse and sustainablel and use system, coping with land shortages	Sub-Saharan Africa South	[7,9]
Grow in low fertile soil	Can be harvested within short periods of time after planting	South Africa	[89]
ITFCs are exchanged, selected, and conserved by farmers who want to promote hybrid vigor and maintain yields	This is also of interest as it promotes a greater variety of size, shape, taste, appearance, adaptability, and maintains biodiversity of ITFCs	Africa	[109]
ITFCs contribute to conservation efforts, preservation, and enhancement of biodiversity	ITFCs and the gardens in which they develop help with ecosystem restoration as well as the conservation of both threatened and commercially valuable indigenous plants species	South Africa	[14,108]

A means to supply urban dwellers with fresh food with a low carbon footprint	Especially in emerging urban communities	Cape Town, South Africa	[106]
Home gardens provide a refuge for ITFCs, which are constantly eradicated due to their undervaluation	From the perspective of urbanization and deforestation	Africa	[10]

**Table 4:** Socio-cultural benefits of ITFCs.

Socio-cultural benefits of ITFCs			

Description	Findings	Location	Reference
Traditional food habits expressed and reinforced by holistic utilization.	Allows for the strengthening of cultural identity, community development, and collective heritage.	Africa	[31,41,104,120]
ITFCs can be a source of novelty food.	Particularly for specialty restaurants catering for the tourist trade.	Southern Africa	[105]
Food processing cooperative within urban regions using ITFCs would contribute towards promoting quality of life.	Increased quality of life in a variety of ways, including income, jobs, and greater demand for ITFCs.	Limpopo, South Africa	[121]
Close connection to land due to the awareness of ITFCs creates continued adaptive management.	This knowledge and understanding are encoded into stories, norms, rules, and institutions.	Africa	[10]
The manifestation of indigenous food sovereignty has developed its own definition of policy and rights.	Therefore allowing for a greater awareness and power associated with ITFCs.	Eastern Cape, South Africa	[78]
ITFCs can strengthen the role of women’s identity.	Women care for and cultivate ITFCs, therefore making a lot of money, and so improving their position in society.	Benin	[122]
ITFCs are interlinked with indigenous food systems.	This represents sustainable livelihoods, biodiversity conservation, and traditions. An approach that could play an important role in addressing global food requirements.	Africa	[123,124]
ITFCs and the gardens where they occur help with social upliftment and crime reduction.	By enhancing and strengthening social contact, although this is not necessarily experienced equally.	Cape Town, South Africa	[106]
An efficient interaction between local knowledge and the nutritional value of ITFCs.	ITFCs and their food systems create a balance between nature and culture.	Africa of Africa	[10,125]
The upsurge in ethnobotanical studies adds impetus.	Revitalizing the use of ITFCs.	Eastern Cape, South Africa	[126]
ITFCs may provide a basis for local seed banks.	Creation of farmer seed autonomy.	Sub-Saharan Africa	[9]
ITFCs contribute to promoting healthy environments	For people’s inner wellness, offering psychological benefit.	Africa	[55]
Food tourism is of increasing relevance	Local culture becomes a tourism resource using ITFCs and encourages adventurous chefs and entrepreneurs to invest in local cuisine. ITFCs therefore will enhance local community “brand identity”.	South Africa	[41,76]
The use of documentation of ITFCs helps preserve the knowledge and prevents a loss of valuable information	This would make a large contribution to literature and knowledge, benefiting communities through easy access to information on the medicinal uses and allowing greater awareness of ITFCs.	Benin, Ghana	[122]
ITFCs provide a “hidden harvest”	Use of ITFCs as co-evolving species to supplement both earnings and food.	Africa	[10]
Cultures are adapted to localities and so offer greater resilience	Therefore, communities are configured to a range of livelihoods and land use which is best suited for their resources and capabilities	Africa	[10]

**Table 5:** Economic benefits of ITFCs.

Economic benefits of ITFCs			

Description	Findings	Location	Reference
Trading with ITFCs can result in employment	Therefore serving as a pivot to increase household income and enhance local economy.	Africa	[10]
	ITFCs can provide substantial value worth R170 into households’ monthly income	Benin, South Africa and Tanzania	[41,51,76,79]
ITFCs as a commodity are often cheaper than exotic counterparts	Increased household savings	Kenya	[78]
ITFCs help raise tax revenue	This is due to general commerce	Africa	[55]
ITFCs can create access points into informal markets	ITFCs have low entry and exit cost and simple mechanisms to sell surplus produce	SADC region	[34]
The health effects known of certain ITFCs may provide a potential cost-competitive source as raw material from primary producers	Usage as functional foods (functional food here refers to food that contains health-giving additives)	Africa	[140]
