# Supplementary material for: A Review of Indigenous Food Crops in Africa and the Implications for more Sustainable and Healthy Food Systems
Source: Sustainability. Author manuscript; Available in PMC 2021 Jan 29. (PMC7116648; doi:10.3390/su12083493)
Supplement: Appendix [file EMS110474-supplement-Appendix.pdf]

Appendix

Appendix 1. ITFCs have high macro and micro-nutrient value.

Macro and Micro-Nutrient value of ITFCs

Type of ITFC	Nutrient	Distribution	Reference
African oil bean seed *(Pentaclethra macrophylla)*	Calcium, Phosphorus	West Africa	[147]
African eggplant/Garden egg *(Solanum melongena; Solanum aethiopicum)*	Vitamin A, (B2)/Riboflavin, Vitamin C, Calcium, Iron, Potassium	Sub-Saharan Africa	[91,148]
African nightshade *(Solanum scabrum)*	Iron, Potassium, Beta-carotene	East and Southern Africa	[50,89,149,150]
African pear/Safou *(Dacryodes edulis)*	Potassium, Calcium, Magnesium	West and Central Africa	[148]
African wild potato, Native potatoes (*Hypoxis hemerocallidea*), *(Solenostemon rotundifolius)*, *(Plectranthus esculentus)*	Calcium, Vitamin A, Iron	West Africa, East Africa and Southern Africa	[91,148]
African breadfruit *(Treculia afriicana)*	Calcium, Potassium, Magnesium	West Africa	[151]
African pearwood, Djave nut, or Moabi *(Aillonella toxisperma)*	Calcium, Magnesium, Phosphorus	West Africa	[147]
Aizen *(Mukheit)*	Zinc, Calcium, Phosphorus, Iron, B Vitamins		[37,55]
Amaranth *(Amaranthus spp.)*	B2/Riboflavin, Vitamin A, Vitamin C, Manganese, Copper, Vitamin K, Potassium, Zinc, Iron,	Africa	[30,50,89,149,152,153]

Macro and Micro-Nutrient value of ITFCs			
**Type of ITFC**	**Nutrient**	**Distribution**	**Reference**
	Calcium, Magnesium, Phosphorus		
Bambara groundnut *(Vigna subterranea)*	Calcium, Iron	West Africa, Central African Republic and Southern Africa	[8]
Balsam apple *(Momordica balsamina)*	Calcium, Magnesium, Iron	Southern Africa	[2]
Baobab (*Adansonia digitata*)	B2/Riboflavin, Calcium, Phosphorus, Iron, Vitamin A, Vitamin C	Sub-Saharan Africa	[55,91,148]
Bird plum *(Berchemia discolour)*	Protein, Fiber	East and Southern Africa	[154]
Bitter leaf *(Vernonia amygdalina)*	Sodium, Potassium, Calcium, Magnesium, Zinc, Iron	West Africa	[155]
Black jack *(Bidens pilosa)*	Copper, Magnesium	Southern Africa	[2]
Black nightshade *(Solanum nigrum)*	Calcium, Magnesium, Iron	East and Southern Africa	[2]
Bush apple *(Heinsia crinita)*	Calcium, Magnesium, Potassium, Iron, Zinc	West, South and Central Africa	[156,157]
Bush mango/Dika *(Irvingia gabonensis), (Irvingia wombulu*	Vitamin C, Vitamin A, Potassium, Calcium, Phosphorus	West Africa	[55,91,158]
Butterfruit *(Persea Americana)*	Sodium, Manganese, Copper, Zinc, Potassium, Magnesium	Africa	[55]
Cassava leaves *(Manihot esculenta)*	Vitamins B1, B2, C, carotenoids, phosphorous, Magnesium, Potassium, Calcium	Sub-Saharan Africa	[159]
Cat's whiskers *(Orthosiphon aristatus)*	Beta-carotene, Magnesium, Iron, Phosphorus, Calcium	South Africa	[149]
Celosia *(Celosia argentea)*	Calcium, Phosphorus, Iron, Calcium, Magnesium	Nigeria, Benin, and Congo,	[91]
Cowpea *(Vigna unguiculata)*	Iron, Zinc, Vitamin A, Vitamin C	West, East, Central and Southern Africa	[8,89,91,148]
Cowpea leaves *(Vigna unguiculata)*	Magnesium, Phosphorus, Calcium	West, East, Central and Southern Africa	[149]
Dry bean *(Phaseolus vulgaris)*	Iron, Zinc	East and Southern Africa	[8]
Egusi *(Citrullus lanatus)*	B1, B2/ Riboflavin, Niacin, Phosphorus, Potassium, Magnesium, Manganese, Sulfur, Calcium, Iron, Zinc, Vitamin A	West Africa	[89,91,149]
Emmer *(Triticum dicoccum)*	Calcium	Sub-Saharan Africa	[160]
Enset *(Ensete ventricosum)*	Calcium	Ethiopia, Nigeria, Sudan, South Africa	[91]
Finger millet *(Eleusine coracana)*	Calcium, Phosphorus, Magnesium, Potassium, Iron, manganese	Burundi, Rwanda, and Zaire, Uganda and Ethiopia	[161]
Fonio *(Digitaria exilis; Digitaria iburua)*	Phosphorus	West Africa	[148]

Macro and Micro-Nutrient value of ITFCs			
**Type of ITFC**	**Nutrient**	**Distribution**	**Reference**
Forest num-num *(Carissa bispinosa)*	Calcium, Phosphorus, Vitamin C, Magnesium	South and East Africa	[37,55,162]
Icacina *(Icacina oliviformis);Icacina senegalensis)*	Magnesium, Calcium, Phosphorus, Potassium	West and Central Africa	[163]
Lablab *(Lablab purpureus)*	Iron	Egypt, Sudan East Africa	[91]
Lasianthera africana	Calcium, Magnesium, Potassium, Iron, Zinc	West and Central Africa	[156,157]
Locust bean *(Parkia biglobosa)*	Calcium, Iron	Togo, Ghana, Burkina Faso, and Nigeria	[91,148]
Long bean *(Vigna unguiculata)*	Vitamin A, Folate	Uganda and Tanzania	[91]
Marama bean *(Tylosema esculentum)*	Potassium, Phosphorus, thiamin, B2/Riboflavin, nicotinic acid	Southern Africa (Angola, Namibia, Botswana and South Africa)	[91]
Marula *(Sclerocarya birrea)*	Calcium, Zinc, Magnesium, Vitamin C, Phosphorus, Potassium	Southern Africa	[55,148]
Monkey orange *(Strychnos spinosa)*	Vitamin C, B Vitamins	Subtropical Africa	[55,148]
Moringa *(Moringa oleifra)*	pro Vitamin A, Folate, Calcium, Iron, Vitamin C, Potassium	Sub-Saharan Africa	[91,148]
Nettle *(Urtica urens)*	Manganese, Iron, Zinc	East and Southern Africa	[164,165]
Pearl millet *(Pennisetum glaucum)*	Potassium, Phosphorus, Trace elements, Vitamin A	Sub-Saharan Africa	[7]
Pigeonpea *(Cajanus cajan)*	Protein	West Africa, East Africa and Southern Africa	[148]
Pigweed *(Chenopodium album)*	Vitamin C, Iron, Zinc, Magnesium	Southern Africa	[2]
Pumpkin leaves *(Cucurbita spp)*	Beta-carotene, Iron, Phosphorus	South Africa	[149]
Rapeseed *(Brassica napus, Brassica oleracea)*	Iron, Zinc, Potassium	East and Southern Africa	[165]
Red-milkwood *(Mimusops caffra)*	Calcium, Potassium, Magnesium, Sodium	South Africa	[166]
Safou *(Dacryodes edulis)*	Calcium		[55]
Spider plant *(Cleome hassleriana); (Cleome gynandra)*	Iron, Vitamin A, Beta-carotene, Vitamin C	East and Southern Africa	[2,30,89]
Sweet potato (tuber) *(Ipomea batatas)*	Beta-carotene	Africa	[7]
Sweet potato leaves *(Ipomea batatas)*	Calcium, Magnesium, Potassium, Iron, Zinc	Sub-Saharan Africa	[157]
Tallow fruit *(Detarium microcarpum)*	Potassium, Phosphorus, Magnesium, Vitamin C, Vitamin E	West and Central Africa	[167]
Tamarind (*Tamarindus indica*)	(B2)/Riboflavin, Calcium, Iron, Potassium		[55]
Taro/Cocoyam (leaves) *(Colocasia esculenta)*	Calcium, Magnesium, Potassium, Iron, Zinc	Africa	[156,157]

Macro and Micro-Nutrient value of ITFCs			
**Type of ITFC**	**Nutrient**	**Distribution**	**Reference**
Taro/Cocoyam (tuber) *(Colocasia esculent)*	Vitamin A, Vitamin C	Sub-Saharan Africa	[7]
Teff *(Eragrostis tef)*	Calcium, Phosphorus, Magnesium, aluminum, Iron, copper, zinc, boron, barium, thiamin	Ethiopia, Eritrea	[148]
Tree grapes *(Jabuticaba)*	Zinc		[55]
Water leaf *(Talinum triangulare)*	Beta-carotene,	Nigeria	[105]
Weeping love grass *(Eragrostis curvula)*	Iron, Magnesium	Eritrea and Ethiopia	[73]
Wild cucumber *(Cucumis africanus)*	Calcium, Magnesium	Southern Africa	[168]
Wild jute *(Corchorus tridens)*	Magnesium, Phosphorus, Calcium, Iron	South Africa	[149,165]
Wild mustard *(Sinapis arvensis)*	Calcium, Iron	Sub-Saharan Africa	[7]

Appendix 2. ITFCs have high energy content, proteins, and dietary fiber.

ITFCs provide Energy, Proteins, and Fiber			
**Type of ITFC**	**Nutrient**	**Distribution**	**Reference**
African oil bean seed *(Pentaclethra macrophylla)*	Energy and caloric contributor, lipids, dietary fiber	West Africa	[169]
African fan palm *(Borassus aethiopum)*	Lipids	Africa	[170]
African nightshade *(Solanum scabrum)*	Protein	Africa	[50]
African pear/Safou *(Dacryodes edulis)*	Fatty acids, proteins	West and Central Africa	[148]
African wild potato, Native potatoes (*Hypoxis hemerocallidea*), *(Solenostemon rotundifolius)*, *(Plectranthus esculentus)*	Energy and caloric contributor, protein,	West Africa, East Africa and Southern Africa	[91,148]
African yam beans (legume) *(Sphenostylis stenocarpa)*	Energy and caloric contributor, dietary fiber, protein	Nigeria	[91]
African yam beans (tuber) *(Sphenostylis stenocarpa)*	Energy and caloric contributor, dietary fiber, protein	Côte d'Ivoire, Ghana, Togo, Cameroon, Gabon, Democratic Republic of Congo, Ethiopia, Malawi and Zimbabwe	[91]
African breadfruit *(Treculia afriicana)*	Protein, energy and caloric contributor	West Africa	[151]
Amaranth *(Amaranthus spp)*	Energy and caloric contributor, protein, dietary fiber, dietary fiber, fatty acids, moisture	Africa	[7,50,59,149]
Balanites *(Balanites aegyptiaca)*	Protein		[55]
Balsam apple *(Momordica balsamina)*	Protein	Southern Africa	[2]

ITFCs provide Energy, Proteins, and Fiber			
**Type of ITFC**	**Nutrient**	**Distribution**	**Reference**
Bambara groundnut *(Vigna subterranea)*	Protein, energy and caloric contributor, fatty acids	West Africa, Central African Republic and Southern Africa	[30,91,148]
Baobab (*Adansonia digitata*)	Energy and caloric contributor, protein, dietary fiber	Sub-Saharan Africa	[55,91]
Bird Plum *(Berchemia discolor)*	Protein, dietary fiber	East and Southern Africa	[154]
Bitter leaf *(Vernonia amygdalina)*	Moisture	West Africa	[155]
Black jack *(Bidens pilosa)*	protein, dietary fiber	Southern Africa	[2]
Black nightshade *(Solanum nigrum)*	Dietary fiber	East and Southern Africa	[2]
Bush apple *(Heinsia crinita)*	Dietary fiber	West South and Central Africa	[156,157]
Butterfruit *(Persea Americana)*	Energy and caloric contributor, fatty acids	Africa	[55]
Cassava (tuber) *(Manihot esculenta)*	Energy and caloric contributor	Sub-Saharan Africa	[55]
Cassava leaves *(Manihot esculenta)*	Amino acids	Sub-Saharan Africa	[159]
Celosia *(Celosia argentea)*	Protein, moisture, dietary fiber	Nigeria, Benin, and Congo,	[91]
Cowpea *(Vigna unguiculata)*	Energy and caloric contributor, protein, dietary fiber	West, East, Central and Southern Africa	[8,59,89,148,149]
Dika (*Irvingia wombolu), (Irvingia gabonensis)*	Energy and caloric contributor, fatty acids, proteins	West Africa,	[37]
Durum wheat *(Triticum durum)*	Protein, energy and caloric contributor	sub-Saharan Africa	[154]
Egusi *(Cucumeropsis mannii)*	Fatty acids, proteins, dietary fiber	West Africa,	[89,91,148]
Emmer *(Triticum dicoccum)*	Energy and caloric contributor	sub-Saharan Africa	[160]
Enset *(Ensete ventricosum)*	Energy and caloric contributor	Ethiopia, Nigeria, Sudan, South Africa	[91]
Eragrostis *(Eragrostis curvula)*	Dietary fiber	Eritrea and Ethiopia	[73]
Finger millet *(Eleusine coracana)*	Energy and caloric contributor, protein	Ethiopia, Uganda, Sudan	[148]
Fluted pumpkin *(Telfairia occidentalis)*	Fatty acids, proteins	West and Central Africa	[122]
Fonio *(Digitaria exilis)* and *Digitaria iburua)*	Energy and caloric contributor, protein, dietary fiber	West Africa	[148,153]
Garden egg *(Solanum melongena)*	Moisture, protein, dietary fiber	West, East, Central and Southern Africa	[91]
Gingerbread plum *(Parinari spp, Neocarya marcophylla)*	Fatty acids	Niger, Guinea, Senegal, Madagascar	[55,171]
Groundnut *(Arachis hypogaea)*	Fatty acids	Africa	[8]
Hausa groundnut *(Macrotyloma geocarpum)*	Protein	West and Central Africa	[122,156]

ITFCs provide Energy, Proteins, and Fiber			
**Type of ITFC**	**Nutrient**	**Distribution**	**Reference**
Icacina *(Icacina oliviformis; Icacina senegalensis)*	Protein, energy and caloric contributor	West and Central Africa	[163]
Icacina *(Icacina trichantha)*	Energy, fatty acids		[55]
Jew's mallow (*Corchorus olitorius)*	Dietary fiber	South Africa	[59]
Lablab *(Lablab purpureus)*	Energy and caloric contributor, protein	Egypt, Sudan East Africa	[91,148]
Lasianthera africana	Dietary fiber	West and Central Africa	[156,157]
Locust bean *(Parkia biglobosa)*	Energy and caloric contributor, protein	Togo, Ghana, Burkina Faso, and Nigeria	[91,148]
Long bean *(Vigna unguiculata)*	Dietary fiber, fatty acids, protein,		[91]
Marama bean *(Tylosema esculentum)*	Energy and caloric contributor, protein, fatty acids	Southern Africa	[91,148]
Marula *(Sclerocarya birrea)*	Energy and caloric contributor, protein, fatty acids	Southern Africa	[55,148]
Monkey orange *(Strychnos spinosa)*	Energy and caloric contributor	Subtropical Africa	[55,148]
Moringa *(Moringa oleifra)*	Protein	Sub-Saharan Africa	[91]
Pearl millet *(Pennisetum glaucum)*	Energy and caloric contributor, protein, dietary fiber	West Africa and East Africa	[172]
Pigeonpea *(Cajanus cajan)*	Protein	West Africa, East Africa and Southern Africa	[148]
Pigweed *(Chenopodium album)*	Protein	Southern Africa	[2]
Raapuintjie *(Cyanella hyacinthoides)*	Protein	South Africa	[105]
Rapeseed *(Brassica napus, Brassica oleracea)*	Dietary fiber	East and Southern Africa	[165]
Senna *(Senna occidentalis)*	Protein	Sub-Saharan Africa	[68]
Spider plant *(Cleome hassleriana)*	Protein, dietary fiber	North West, East and Southern Africa	[14,50,59]
Sweet detar *(Detarium senegalense)*	Energy		[55]
Sweet potato (leaves) *(Ipomea batatas)*	Dietary fiber	West Africa, Southern Africa	[157]
Tallow (*Detarium microcarpum)*	Energy and caloric contributor	West and Central Africa	[167]
Tamarind (*Tamarindus indica*)	Caloric contributor	Sub-Saharan Africa	[55]
Taro/Cocoyam (leaves) *(Colocasia esculenta)*	Dietary fiber	Africa	[156,157]
Taro/Cocoyam (tuber) *(Colocasia esculent)*	Energy and caloric contributor	Africa	[173]
Teff *(Eragrostis tef)*	Energy and caloric contributor, protein	Ethiopia, Eritrea	[148]
Tigernuts *(Cyperus esculentus)*	Proteins	West and Central Africa	[122]
Tree grapes *(Jabuticaba)*	Dietary fiber		[55]
Nettle *(Urtica urens)*	Protein, dietary fiber	East and Southern Africa	[165]
Water leaf *(Talinum triangulare)*	Dietary fiber	Nigeria	[105]

**ITFCs provide Energy, Proteins, and Fiber**			
**Type of ITFC**	**Nutrient**	**Distribution**	**Reference**
Watermelon *(Citrullus lanatus var. lanatus)*	Dietary fiber, moisture	Africa	[55]
Wild cucumber *(Cucumis africanus)*	Moisture	Southern Africa	[168]
